# Phytobezoar: A Brief Report with Surgical and Radiological Correlation

**DOI:** 10.1155/2018/5253162

**Published:** 2018-03-26

**Authors:** Sameer A. Hirji, Faith C. Robertson, Grace F. Chao, Bharti Khurana, Jonathan D. Gates

**Affiliations:** ^1^Department of Surgery, Brigham and Women's Hospital, Harvard Medical School, Boston, MA, USA; ^2^Harvard Medical School, Boston, MA, USA; ^3^Department of Surgery, Yale-New Haven Hospital, Yale School of Medicine, New Haven, CT, USA; ^4^Department of Radiology, Brigham and Women's Hospital, Harvard Medical School, Boston, MA, USA; ^5^Department of Surgery, Hartford Hospital, University of Connecticut, Hartford, CT, USA

## Abstract

Gastrointestinal bezoars, collections of incompletely digested material within the alimentary tract, can present as a diagnostic challenge and should be considered in the differential diagnosis and management of small bowel obstruction, ischemic bowel, or bowel perforation. We present a case of a 37-year-old man with a distant history of laparotomy for superior mesenteric artery thrombosis requiring partial small bowel resection of the jejunum who presented with worsening abdominal pain, nausea, vomiting, and hematemesis. An abdominal computed tomography revealed dilated loops of small bowel with a transition point at the ileum, distal to his prior bowel anastomosis. He was managed initially nonoperatively, but persistent vomiting and worsening distention necessitated urgent exploratory laparotomy. During the procedure, a 4 cm by 3 cm phytobezoar was discovered at the midjejunum. The patient had an unremarkable postoperative course with no further symptoms at 1-year follow-up. Timely diagnosis and treatment of bezoar is essential to minimize patient complications.

## 1. Introduction

Gastrointestinal bezoars are rare but often can present with serious complications if untreated. While the physical nature of these bezoars varies, the extent to which they can affect the motility of the alimentary tract is also variable, creating a challenge both in diagnosis and management. In this case, we present the clinical, radiographic, and operative findings of a man who presented with a phytobezoar in the setting of a small bowel obstruction. We also review the existing literature to address the appropriate diagnostic and treatment strategies to minimize perioperative complications.

## 2. Case Presentation

**Figure 1:**
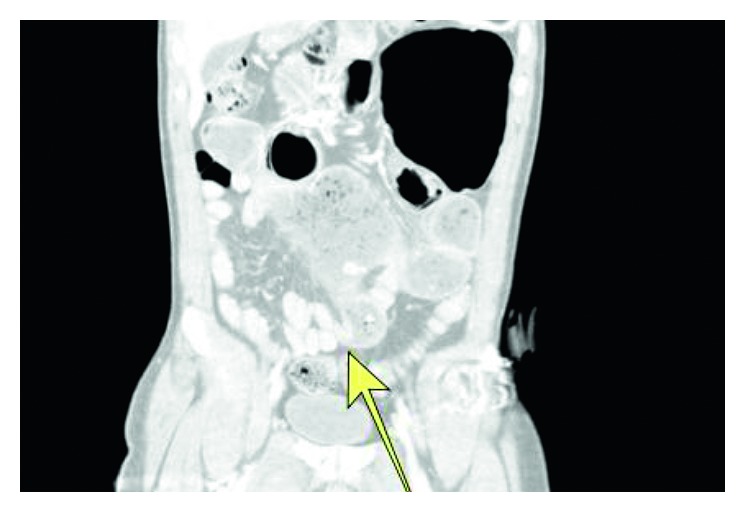
Computed tomography image demonstrating the small bowel obstruction secondary to the phytobezoar. A transition point is seen in the midline lower abdomen in the region of the proximal ileum, with distended proximal small bowel loops containing fecalized material and decompressed distal small bowel loops (yellow arrow). There is a similar appearance of the distended left upper quadrant bowel anastomosis site, as well as within the side-to-side small bowel anastomosis in the midabdomen. A small amount of mesenteric fluid is seen, without significant ascites, small bowel wall thickening, differential mucosal enhancement, or pneumatosis to indicate bowel compromise.

The patient is a 37-year-old man with a past medical history of hypertension, diabetes mellitus, extensive hypercoagulable history, and a remote history of exploratory laparotomy for an embolectomy of the superior mesenteric artery and a small bowel resection of the jejunum. He presented to the Emergency Department with an 8-hour history of worsening, crampy abdominal pain, nausea, vomiting, and multiple episodes of hematemesis. He had a bowel movement prior to arrival, but otherwise his review of systems was unremarkable. Physical examination was notable for hemodynamic stability and a mildly distended abdomen with epigastric tenderness but without guarding or rebound tenderness. Given his prior surgical history, a computed tomography (CT) of the abdomen was obtained, which showed dilated loops of bowel with a transition point in the region of the proximal ileum, just distal to his prior bowel anastomosis. The distended proximal small bowel loops contained fecalized material, and the distal small bowel loops were also decompressed without evidence of ascites, bowel wall enhancement, or free air (Figure 1).

**Figure 2:**
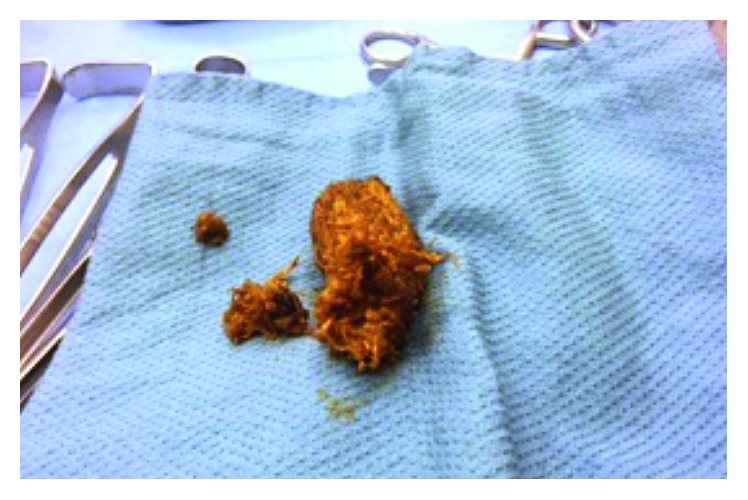
Intraoperative photograph of the bezoar. A longitudinal enterotomy was performed to evacuate the phytobezoar, which measured 4 cm by 3 cm in diameter, and consisted of fibrous vegetable matter and acellular debris.

The patient was admitted and initially managed nonoperatively with fluid resuscitation, pain control, bowel rest with nasogastric tube decompression, and serial abdominal exams. Following return of bowel function, the nasogastric tube was discontinued on hospital day 2 but reinserted hours later due to persistent, new-onset vomiting, and worsening abdominal distention. Without significant improvement in symptoms, the patient was taken to the operating room urgently for an exploratory laparotomy. Intraoperatively, the small bowel was dilated to approximately the midjejunum, where an intraluminal obstruction was palpated and subsequently identified to be a phytobezoar. Consequently, a longitudinal enterotomy was performed to evacuate the phytobezoar measuring 4 cm by 3 cm and was closed in a transverse fashion in two layers (Figure 2). The phytobezoar seemed to be composed of fibers, but material resembling skins and seeds were not identified. There was also no evidence that this obstruction was located at the suture line, and no other intraluminal pathology was noted that would have narrowed the lumen of the bowel. Final pathology revealed “vegetable matter and acellular debris.” On further questioning the patient, the patient endorsed a diet prior to hospitalization that was significant for large amounts of vegetables. However, because no finer detail was elicited, further conclusions about the content could not be drawn. The patient continued to progress well postoperatively with eventual return of bowel function and was discharged home without complications. The patient demonstrated good recovery from his surgery and was asymptomatic at his postoperative clinic visit and at 1-year follow-up.

## 3. Discussion

Gastrointestinal bezoars are collections of incompletely digested material within the alimentary tract that can lead to serious medical complications including small bowel obstruction, bowel ischemia, or perforation. Bezoars are classified by their contents: phytobezoars involve fibrous nondigestible matter from fruit, vegetables, psyllium, or persimmon (diospyrobezoar); trichobezoars comprised ingested hair, prevalent in women aged less than 30 years; pharmacobezoars arise from enteric-coated aspirin, extended release capsules (nifedipine and theophylline), or sucralfate; and occasionally, bezoars result from ingested styrofoam, fungi, cement, or other inedible objects. While phytobezoars are the most common types of bezoar, phytobezoar-induced small bowel obstruction is a rare clinical situation, accounting for less than 5% of all mechanical intestinal obstructions [1].

Patients with a history of peptic ulcer disease, previous gastric surgeries or bowel resections, and systemic diseases that reduce gastrointestinal motility or cause strictures (e.g., Crohn's disease, carcinoma of the gastrointestinal tract, chronic dehydration, hypothyroidism, diabetes, and in some cases, connective tissue-related disorders) are predisposed to bezoar formation [2]. Excessive vegetable fiber ingestion or alterations in dentition and mastication can also increase one's risk for complications as the ability to distinguish nutritionally valuable material from that which is not is compromised. Consequently, phytobezoar should be considered as part of the differential diagnosis for patients with a clinical history, similar to our patient, who presented with symptoms of bowel obstruction or an acute surgical abdomen, especially when no obvious underlining mechanical or physiological causes of obstruction can be identified. Likewise, the presence of bezoars should be considered in patients with an extensive psychiatric history who present with bowel obstruction, although most often, this aspect is ignored during initial patient evaluation, given its relative rarity. Lastly, patients with gastroparesis may also be susceptible to gastric outlet syndrome if they develop a phytobezoar [3].

In patients with small bowel obstruction due to phytobezoar, the ileum is the most common site of obstruction [4]. Two recent case reports also emphasize this finding. In the first case, a 55-year-old male with a previous gastrectomy presented with an ileal phytobezoar causing small bowel obstruction. The second case involved a patient from Iran who presented with a large obstruction at the ileocecal valve [5]. Of note, in the latter case, the patient had eaten 3 kg of persimmons in one sitting two weeks prior to admission. While our patient had no dietary history persimmons consumption, he endorsed a recent diet significant for large amounts of vegetables. Further information on the specifics of his diet was lacking, but we suspect that consumption of large amounts of vegetables rich in fiber (e.g., figs, sauerkraut, brussels sprouts, and potato peel), which are composed of cellulose, hemicellulose, or tannins, could have been the source of the phytobezoar.

Radiographically, phytobezoars are well-circumscribed intraluminal masses that have a heterogeneous appearance, often with a mottled, air-bubble pattern and an absence of mesenteric fat stranding [6]. They can be seen with abdominal radiography, ultrasonography, or CT as a mass or a filling defect. A recent study of bezoars in China by Kuang et al. examined risk factors for small bowel obstruction caused by bezoars and found that major diameter measured on CT scans was a significant independent predictor [7]. In general, bezoars most commonly occur in the distal small intestine or the jejunum and in some cases, are associated with Meckel's diverticula in the ileum [7]. The radiographic appearance of the bezoar on CT scan may be similar to the fecalized small bowel content seen with established small bowel obstructions (Figure 2). Furthermore, there are no reports of phytobezoar-induced ileus, to our knowledge, that was diagnosed preoperatively. Thus, preoperative diagnosis of phytobezoar is unlikely as opposed to small bowel obstruction. In our patient, definite diagnosis of phytobezoar prior to surgical removal was not possible and perhaps also not possible retrospectively given the nonspecific appearance of fecalized small bowel content and the limited ability of CT scans to distinguish finer details of the mass. Notably, our patient had no obvious areas of intrinsic bowel stenosis, and this location of the bezoar was more distal than the prior bowel anastomosis and more proximal than the natural narrowing at the level of a Meckel's diverticulum. The rare midjejunal location of the obstruction in our patient likely was a combination of intra-abdominal adhesions and the continual accretions of the phytobezoar.

Diagnosis of bezoar is often delayed, but once established, there are several treatment options available. Medically, treatment with prokinetic agents such as itopride, mosapride, and metoclopramide or enzymatic breakdown (dissolution) is often sufficient. If the lesion is confined to the stomach or duodenum, endoscopic fragmentation with forceps or snares can be employed to diagnose and deconstruct the bezoar with a goal of alleviating the bowel obstruction. Additionally, for gastric bezoars, Coca-Cola has been shown to be an effective dissolution agent [8]. For those with failure to progress or worsening clinical symptoms, surgery is the gold standard method to avoid bowel ischemia and bezoar-induced pressure necrosis. This almost always involves an exploratory laparoscopy or laparotomy with bowel enterotomies with or without primary resection. Intraoperatively, the bezoar may be fragmented and “milked” to the cecum or resected via enterotomy.

Timely diagnosis and treatment of bezoar is essential to minimize patient complications, although in most scenarios, this task is difficult to accomplish. In the current era, the utility and dependence of imaging to aid in the diagnosis cannot be underestimated. Therefore, bezoar obstruction, although rare, should be an important consideration, especially during the initial evaluation and workup of patients presenting with abdominal symptoms to the emergency room.
